# Co‐expression of HSV‐1 ICP34.5 enhances the expression of gene delivered by self‐amplifying RNA and mitigates its immunogenicity

**DOI:** 10.1002/2211-5463.70036

**Published:** 2025-04-09

**Authors:** Xuemin Lu, Yabin Wu, Chunye Zhao, Jie Zheng, Shangwu Chen, Yigang Wang, Yulong Xia

**Affiliations:** ^1^ College of Life Sciences and Medicine Zhejiang Sci‐Tech University Hangzhou China; ^2^ Wenzhou Institute University of Chinese Academy of Sciences Wenzhou China; ^3^ College of Pharmacy Zhejiang Chinese Medical University Hangzhou China

**Keywords:** gene expression, ICP34.5, innate immune response, saRNA, vaccines

## Abstract

Self‐amplifying RNA (saRNA) vectors have garnered significant attention for their potential in transient recombinant protein expression and vaccination strategies. These vectors are notable for their safety and the ability to produce high levels of protein from minimal input templates, offering a promising avenue for gene therapy applications. Despite their advantages, saRNA vectors face a critical challenge in their propensity to trigger a robust innate immune response. The presence of double‐stranded RNA intermediates during saRNA replication activates pattern recognition receptors (PRRs), leading to the activation of protein kinase R (PKR) and interferon (IFN) signaling, which can result in a general translational shutdown within the host cell. To mitigate the stimulatory effects on PRRs and enhance the translation efficiency of saRNA, this study employs the saRNA‐encoding HSV‐1 neurovirulence protein ICP34.5, which is known for its ability to counteract the effects of PKR activation, potentially improving the translation efficiency of saRNA. It was shown that saRNA‐encoding ICP34.5 clearly mediated the eukaryotic initiation factor 2 alpha subunit (eIF2α) dephosphorylation and significant suppression of innate immune responses *in vitro*, leading to enhanced expression of saRNA‐encoded genes. The application of ICP34.5 incorporating saRNA vectors offers a more efficient and cost‐effective solution for the production of proteins and the development of vaccines. This strategy could revolutionize the fields where saRNA utilization is envisioned, particularly in neurotropic disease applications where HSV‐1 proteins may offer additional benefits.

## Graphical abstract

ICP34.5 is one of the most important antihost response proteins. The saRNA‐encoding HSV‐1 neurovirulence protein ICP34.5 clearly mediated the eukaryotic initiation factor 2 alpha subunit (eIF2α) dephosphorylation and significant suppression of innate immune responses *in vitro*, leading to enhanced expression of the saRNA‐encoded gene.

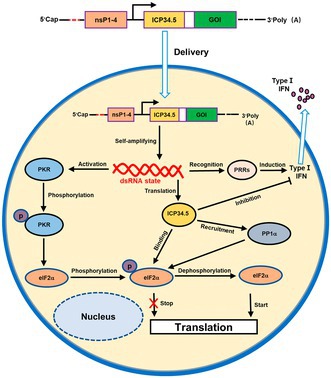

**Abbreviations**
5mC: 5‐methylcytidine
DMEM: dulbecco's modified eagle medium
eIF2α: eukaryotic initiation factor 2 alpha subunit
FBS: fetal bovine serum
GOI: gene of interest
HEK293T: human embryonic kidney
HSV‐1: Herpes Simplex Virus type 1
IFN: interferon
IIP: innate inhibitory proteins
MFI: mean fluorescence intensity
PBMCs: peripheral blood mononuclear cells
pDCs: plasmacytoid dendritic cells
PKR: protein kinase R
PP1: protein phosphatase 1
PRRs: pattern recognition receptors
PVDF: polyvinylidene fluoride
RdRp: RNA‐dependent RNA polymerase
saRNA: self‐amplifying RNA
SFV: semliki forest virus
TBK‐1: TANK‐binding kinase 1
UC‐MSCs: umbilical cord‐derived mesenchymal stem cells
VEEV: venezuelan equine encephalitis virus
α‐MEM: minimum essential medium alpha modification

The development of self‐amplifying RNA (saRNA) has introduced a transformative approach to vaccine design and gene therapy [1, 2, 3]. Several saRNA‐based vaccines are currently in clinical trials, demonstrating promising safety profiles and efficacy [4, 5]. Notably, the world's first saRNA vaccine, ARCT‐154, has been approved in Japan for use as a COVID‐19 booster targeting Omicron variants, marking a milestone in saRNA drug development [6]. Derived from alphaviruses such as Venezuelan equine encephalitis virus (VEEV), Semliki Forest virus (SFV), and Sindbis virus, saRNA harnesses the natural ability of these viruses to replicate their genetic material within host cells [7]. A key advantage of saRNA is its capacity for self‐amplification, which results in a high copy number of up to 2 × 10^5^ copies per cell [8]. This characteristic enables the use of significantly lower amounts of saRNA than conventional mRNA to achieve effective gene transfer and protective vaccination [9, 10, 11]. The self‐amplification process is initiated upon transfection, where the encoded replicase polyprotein is translated and interacts with the 5′ and 3′ termini of the genomic RNA, synthesizing complementary genomic RNA copies [12]. These copies serve as templates for the synthesis of novel positive‐stranded, capped, and poly‐adenylated genomic copies and subgenomic transcripts, leading to high production of antigen *in vivo* [13]. In the context of vaccine development, saRNA has demonstrated superiority over nonreplicating mRNA and DNA vaccines in preclinical models, as it can provide very high expression levels and simultaneously induce strong innate immune responses, thereby potentiating immunity [14, 15].

The unique feature of saRNA represents a promising platform for vaccines and gene therapy due to its ability to produce high levels of protein from minute amounts of transfected templates, offering a significant advantage over conventional mRNA in terms of efficacy and dosage requirements [7, 16, 17]. However, compared with mRNA vaccines, saRNA triggers a more intense innate immune response [9], especially activating PKR and IFN signaling, which is the main barrier to its full utilization [18]. This immune response is initiated by pattern recognition receptors sensing double‐stranded RNA intermediates formed during saRNA translation, leading to a translational shutdown that hinders the expression of the gene of interest (GOI) [19]. Recent studies have shown that the incorporation of modified nucleotides, such as 5‐methylcytidine (5mC), into saRNA can suppress the early interferon response and enhance the expression potency of saRNA [4, 20, 21]. Despite these advancements, there are inherent limitations to this approach. Specifically, the progeny saRNA copies that result from the replication of modified saRNA molecules contain wild‐type nucleotides, as the viral RNA‐dependent RNA polymerase (RdRp) utilizes only wild‐type nucleotides for replication. Therefore, while the initial modified saRNA may exhibit enhanced performance, the subsequent rounds of replication yield wild‐type saRNA, which is subject to the same innate immune responses that limit the duration of antigen expression.

In a recent study, researchers explored the use of nonreplicating mRNA encoding vaccinia virus immune evasion proteins [22]. Codelivery of these mRNAs with saRNA significantly reduced PKR activation and IFN‐b upregulation *in vitro* [22]. Researchers also screened the effects of various innate inhibitory proteins (IIP) from different viral sources on the expression of saRNA and found that saRNA cis‐encoded with the PIV‐5V protein and the MERS‐CoV ORF4a protein can effectively enhance the gene expression of saRNA [23]. Herpes Simplex Virus type 1 (HSV‐1) also expresses several viral proteins that are known to inhibit the host's antiviral innate immune response. These proteins target various components of the immune system to facilitate viral replication and evasion of host defenses [24, 25, 26]. Among them, ICP34.5 is one of the most important antihost response proteins with the ability to inhibit the IFN‐inducible kinase PKR and mediate dephosphorylation of eIF2α by recruiting protein phosphatase 1 (PP1) and directing it to specifically dephosphorylate eIF2α [27, 28]. ICP34.5 also dampens IFN production by binding and sequestering TBK‐1, which phosphorylates IRF3 [27]. All the above mechanisms that counteract host shutoff by ICP34.5 may facilitate the expression of GOI delivered by saRNA.

In this study, we aim to evaluate the ability of HSV‐1 ICP34.5 to enhance the translation and expression of saRNA‐encoded genes and to optimize its use in gene delivery systems. ICP34.5 incorporated into saRNA ensures that offspring saRNA have the same properties. This study shows that the saRNA‐encoding ICP34.5 prolongs GOI expression and thereby improves gene delivery applications.

## Materials and methods

### Cell lines

Human embryonic kidney (HEK293T) cells (ATCC) were cultured in Dulbecco's Modified Eagle Medium (DMEM, Cytiva, Marlborough, MA, USA) containing 10% fetal bovine serum (FBS, ExCell Bio, Suzhou, Jiangsu, China) and 1% Penicillin and Streptomycin. Umbilical cord‐derived mesenchymal stem cells (UC‐MSCs) was derived from Wharton's jelly of the umbilical cord and were purchased from Meisen Cell and cultured in Minimum Essential Medium Alpha Modification (α‐MEM, Cytiva) medium supplemented with 10% FBS, 5 ng·mL^−1^ human recombinant bFGF, and 1% penicillin and streptomycin. All these cells were cultured at 37 °C in a 5% CO_2_ incubator.

### Template design and preparation

All saRNA templates took the form of linearized plasmid DNA. All saRNA templates were generated from a plasmid encoding nonstructural proteins 1–4 of the Venezuela equine encephalitis virus (VEEV) using a T7 promoter (promoter sequence, TAATACGACTCACTATAAT). Sequences from VEEV were derived from T7‐VEE‐GFP [29], which was deposited by S. Dowdy (Addgene, Watertown, MA, USA; Plasmid no. 58977). The ICP34.5 gene sequence was found in the complete genome of human HSV‐1 strain KOS (GenBank: JQ673480.1). ICP34.5 was cloned into a plasmid backbone as part of the GOIs (EGFP) with a P2A cleavage site. Recombinant plasmids were cloned in DH5α Escherichia coli, purified using a Plasmid Midiprep Kit (Omega, Norcross, GA, USA), linearized with MluI‐HF (NEB) for 3 h at 37 °C, and purified again using a PCR Purification Kit (Uelandy, Suzhou, Jiangsu, China).

### *In vitro* transcription of saRNA

Linearized plasmid DNA was used as a template for the IVT reaction via the HiScribe T7 High Yield RNA Synthesis Kit (New England Biolabs, Ipswich, MA, USA), with cotranscriptional capping using CleanCap technology (CleanCap AU; Trilink BioTechnology, San Diego, CA, USA), following the manufacturer's instructions. Briefly, DNA samples were supplemented with unmodified ribonucleotide or 5‐methylcytidine (TriLink BioTechnologies) solutions, MEGAscript reagents, and CleanCap AU, and incubated for 2 h at 37 °C. IVT saRNA samples were treated with 2 U·μL^−1^ DNase I for 15 min at 37 °C to remove template DNA. For IVT saRNA cleanup, samples were mixed with RNA Cleanup Binding Buffer and ethanol (> 95%, EtOH) and transferred on silica‐based spin columns (GeneJET RNA Cleanup and Concentration Micro Kit, Thermo Fisher, Waltham, MA, USA). The saRNA concentration was determined using Nanodrop. The purified saRNA was dissolved in nuclease‐free water and stored at −80 °C until further use.

### saRNA transfection

HEK293T cells and UC‐MSC cells were seeded in 24‐well plates. SaRNA was introduced into the cells (~80% confluent) by using the CALNP mRNA *in vitro* (D‐Nano Therapeutics, Beijing, China). The experimental procedures were based on the provided methods. Briefly, the transfection complex was prepared by sequentially mixing 1 μL mRNA solution (1 mg·mL^−1^), 7 μL Reagent A, and 2 μL Reagent B, followed by a 10‐min incubation at room temperature. Next, add the cell culture medium and gently mix to form the RNA transfection complex. The complex was added to the cells and gently mixed. Cells were cultured at 37 °C with 5% CO₂ for more than 24 h prior to analysis.

### Western blotting

Transfected HEK293T and UC‐MSC cells were harvested and lysed. Total protein amounts were quantified by BCA (Beyotime, Shanghai, China). Equal protein amounts were loaded and transferred to polyvinylidene fluoride (PVDF). After blocking, the PVDF was incubated with eIF2α Rabbit mAb (ABclonal, Wuhan, Hubei, China; 1 : 4000 dilution), Phospho‐eIF2α‐S51 Rabbit mAb (ABclonal; 1 : 2000 dilution) and GAPDH Monoclonal Antibody (Biodragon, Beijing, China; 1 : 5000 dilution). Next, PVDF was incubated with HRP‐conjugated secondary antibody (Beyotime; 1 : 2000 dilution). Western blotting results were analyzed digitally. Next, PVDF was incubated with HRP‐conjugated secondary antibody (Proteintech, Rosemont, IL, USA; 1 : 4000 dilution). Western blotting results were analyzed digitally (Bio‐Rad, Hercules, CA, USA; ChemiDoc 12003153). The grayscale values were analyzed using the ImageJ software.

### Quantitative real‐time polymerase chain reaction (qRT‐PCR)

Total RNA was extracted from cells using the GeneJET RNA Purification kit (Thermo Fisher). cDNA was synthesized using the PrimeScript RT reagent Kit with gRNA Eraser (TaKaRa Bio, Kusatsu, Shiga, Japan). Then, quantitative PCR was performed using a SYBR Green Master Mix Kit (TaKaRa Bio), and real‐time PCR was performed using the Applied Bio Systems 7500 Sequence Detection System (Applied Biosystems, Foster City, CA, USA). The primers and probes used for RT‐PCR were as follows: ICP34.5 Forward; 5′‐CATGTGCCAGAATCTGCCTC‐3′, ICP34.5 Reverse; 5′‐GGAGGCAGTCTGAATGGTCT‐3′, GFP Forward; 5′‐GTCCAGGAGCGCACCATCTT‐3′, GFP Reverse; 5′‐AGCTCGATGCGGTTCACCAG‐3′, NSP4 Forward; 5′‐AGAACGGCTAACCGGATCAC‐3′, NSP4 Forward; 5′‐ATATTCAACCAGGTGGCGCA‐3′, GAPDH Forward; 5′‐GTGGTCTCCTCTGACTTCAACA‐3′, GAPDH Reverse; 5′‐CTCTTCCTCTTGTGCTCTTGCT‐3′. The relative fold change was calculated using the $2^{-\Delta \Delta C_{t}}$ method [30].

### Flow cytometry

Flow cytometric assay was utilized to analyze the expression levels of EGFP.

saRNA‐transfected cells were detached by 1xTrypLE (Thermo Fisher, formerly Ambion, Schwerte, Germany). Then, cells were washed twice with staining buffer and resuspended in 200 μL of staining buffer. Fluorescein Isothiocyanate (FITC) channel was used to detect EGFP expression. The data were analyzed using CytoFLEX (Beckman Coulter Life Sciences, Indianapolis, IN, USA). MFI was analyzed using the FlowJo software (Tree Star, Ashland, OR, USA).

### ELISA essay

Supernatant from transfected cells was collected and centrifuged (300 ***g***, 30 min) to remove cell debris for downstream analysis. Human IFNα2 (MULTI SCIENCES, Hangzhou, Zhejiang, China; EK199) and Human IFNβ (MULTI SCIENCES; EK1236) ELISA kits were then used based on provided directions. Briefly, reagents and samples were equilibrated to room temperature prior to use. Standard solutions were prepared by twofold serial dilution, and 100 μL standard solutions and samples were added to the designated wells. After adding 50 μL of Detection Antibody Working Solution, the plate was sealed and incubated at room temperature for 2 h with gentle shaking. Wells were washed six times with wash buffer, followed by the addition of 100 μL Streptavidin‐HRP Working Solution and a 45‐min incubation. After another wash step, 100 μL of substrate solution was added to each well and incubated in the dark for 5–30 min. The reaction was stopped with 100 μL of Stop Solution. Results were calculated based on corrected OD values. Absorbance was measured at 450 nm.

### Statistical analysis

Statistical analyses were performed by Student's *t*‐test or Mann–Whitney *U* test using the graphpad prism 6 software (GraphPad, San Diego, CA, USA). A value of *P* < 0.05 was considered to indicate significance.

## Results

### Successfully generated designed saRNA

**Fig. 1:**
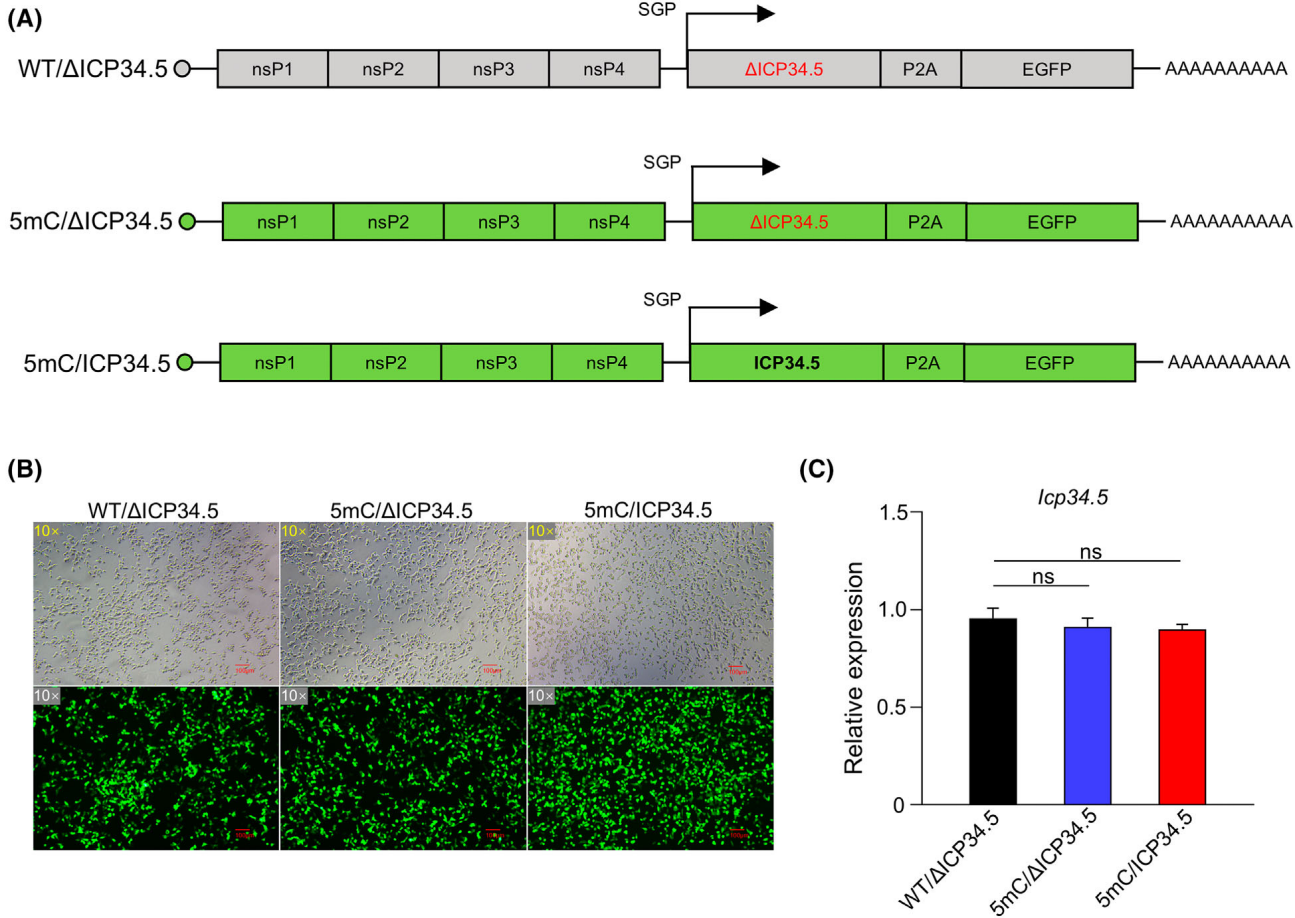
saRNA construction and validation of synthetic saRNA. (A) The structural schematic diagram of wild‐type saRNA, 5mC‐modified saRNA‐encoding delete‐mutated or intact ICP34.5. (B) Representative fluorescence microscopy images of 293T cells transfected with three types of saRNAs at 24 h. (C) The expression of ICP34.5 was identified by qRT‐PCR. Statistical analysis was performed by Student's *t*‐test. Data are means ± SD. *n* = 3. *P < 0.05, **P < 0.01, ***P < 0.001, ns, not significant.

The amino acid sequence spanning positions 218–233 in the ICP34.5 gene is crucial for its binding to PP1, which mediates the dephosphorylation of eIF2α. Consequently, we engineered a deletion mutant of ICP34.5 lacking amino acids 218–233 (ΔICP34.5), thereby disabling its eIF2α dephosphorylation activity, serving as a control (Fig. 1A). As reported, saRNAs modified with 5‐methylcytosine (5mC) can dampen the early antiviral innate immune response and boost gene expression [20, 21]. We synthesized both wild‐type (WT) and 5mC‐modified saRNAs via *in vitro* transcription (IVT) (Fig. 1A). These saRNAs encoded the EGFP reporter gene to assess the effect of fully intact ICP34.5 on protein expression. Given the absence of STAT2 and IRF9 in HEK293 cells, they cannot form a functional ISGF3 complex, leading to a diminished response to Type I interferons and facilitating high‐level protein expression from saRNA [31]. Therefore, we transfected 293T cells with three types of saRNA and monitored EGFP expression under microscopy. We found that all three types of saRNA were able to express high levels of EGFP in 293T cells (Fig. 1B). We also quantified ICP34.5 mRNA at the early phase of transfected cells, finding comparable levels across all samples (Fig. 1C). These findings confirm the successful preparation of WT and 5mC‐modified saRNAs encoding both ICP34.5 and EGFP.

### saRNA‐encoding ICP34.5 mediates eIF2α dephosphorylation

**Fig. 2:**
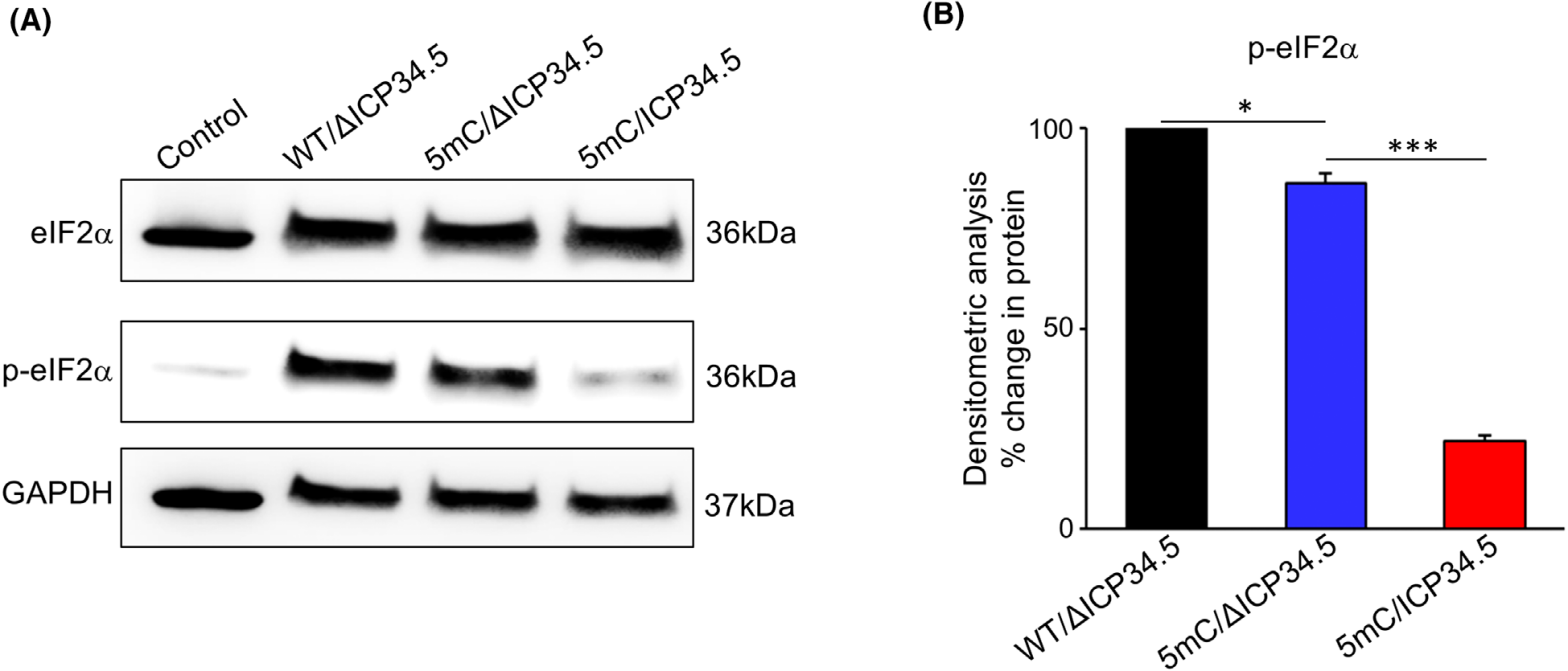
saRNA‐encoding ICP34.5 mediates eIF2α dephosphorylation in UC‐MSCs. (A) Western blot analysis showing the expression levels of eIF2α and phosphorylated eIF2α (p‐eIF2α) in UC‐MSCs transfected with three distinct saRNAs. GAPDH was used as the loading control. (B) After obtaining the western blot bands, grayscale values were analyzed using ImageJ. The intensity of each band was normalized to GAPDH, which served as the internal loading control, to correct for variability in protein loading. The normalized grayscale values of the experimental groups were expressed relative to the wild‐type group, which was set as the baseline (100%). The reduction in signal intensity for the modified groups, calculated relative to the wild‐type, was represented as a percentage decrease. Statistical analysis was performed by Student's *t*‐test. Data are means ± SD. *n* = 3. *P < 0.05, **P < 0.01, ***P < 0.001.

During HSV‐1 infection, the ICP34.5 protein binds to both PP1 and eIF2α, mediating the dephosphorylation of eIF2α and facilitating protein synthesis in host cells. To assess the impact of saRNA‐encoding ICP34.5 on phosphorylated eIF2a levels, we transfected three distinct saRNAs into umbilical cord‐derived mesenchymal stem cells (UC‐MSCs), which possess an intact IFN signaling pathway. Western blot analysis was conducted 24 h post transfection to evaluate the expression levels of eIF2α and its phosphorylated form. We observed that eIF2α expression was consistent across all treatment groups (Fig. 2A). However, 5mC‐modified saRNA induced a modest decrease in eIF2α phosphorylation compared with WT saRNA (Fig. 2A,B). This result indicates that the 5mC‐modified saRNA suppresses the early antiviral immune response, consequently leading to a decrease in the phosphorylation level of eIF2α. Meanwhile, saRNA encoding the intact ICP34.5 protein resulted in even lower phosphorylation levels (Fig. 2A,B). These findings suggest that saRNA‐encoding ICP34.5 is capable of orchestrating the dephosphorylation of eIF2α, thereby hinting at its potential role in augmenting the expression of genes delivered by saRNA within host cells.

### ICP34.5 enhances the expression of saRNA‐encoded genes

**Fig. 3:**
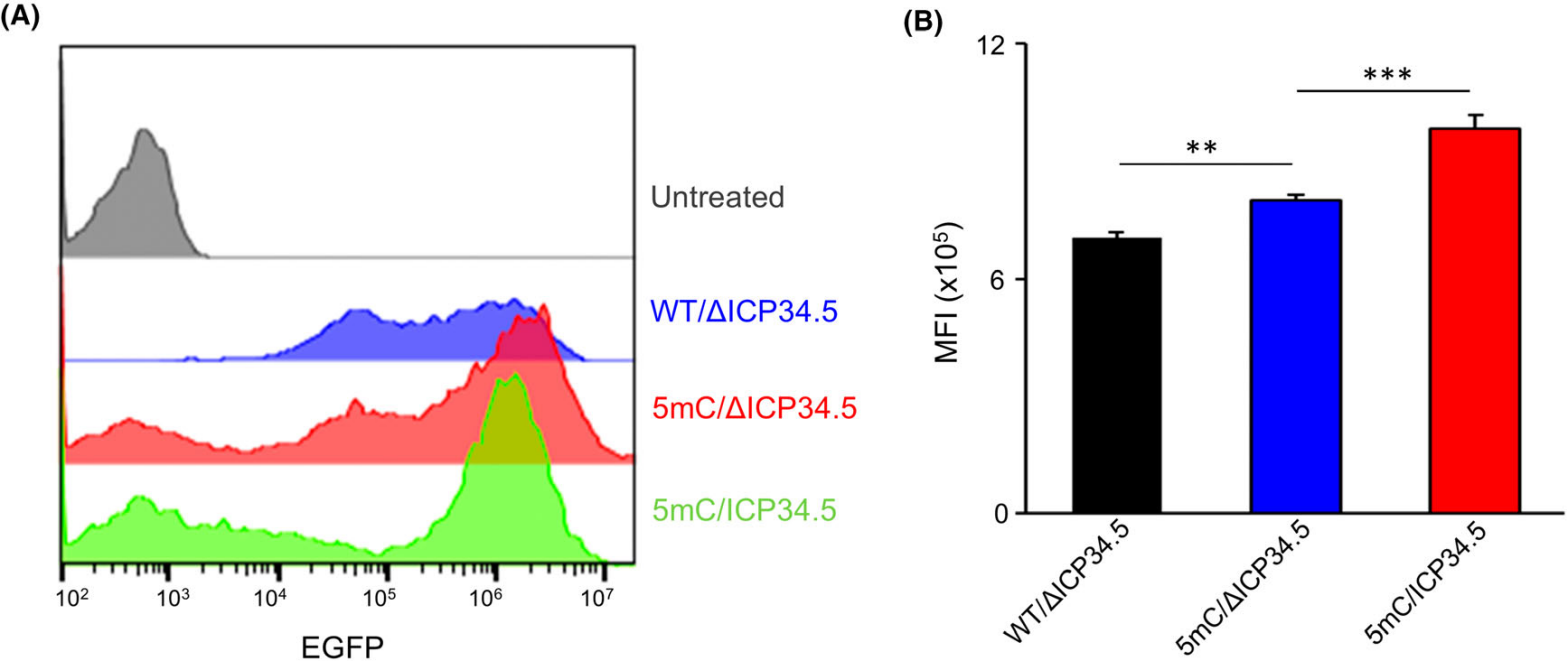
saRNA‐encoding intact ICP34.5 enhances EGFP expression in 293T. (A) Flow cytometry analysis of EGFP expression in 293T cells transfected with three distinct saRNAs for 24 h. (B) Quantification of mean fluorescence intensity (MFI) of EGFP expression in 293T transfected with three distinct saRNAs, based on the flow cytometry analysis shown in (A). Statistical analysis was performed by Student's *t*‐test. Data are means ± SD. *n* = 3. *P < 0.05, **P < 0.01, ***P < 0.001.

**Fig. 4:**
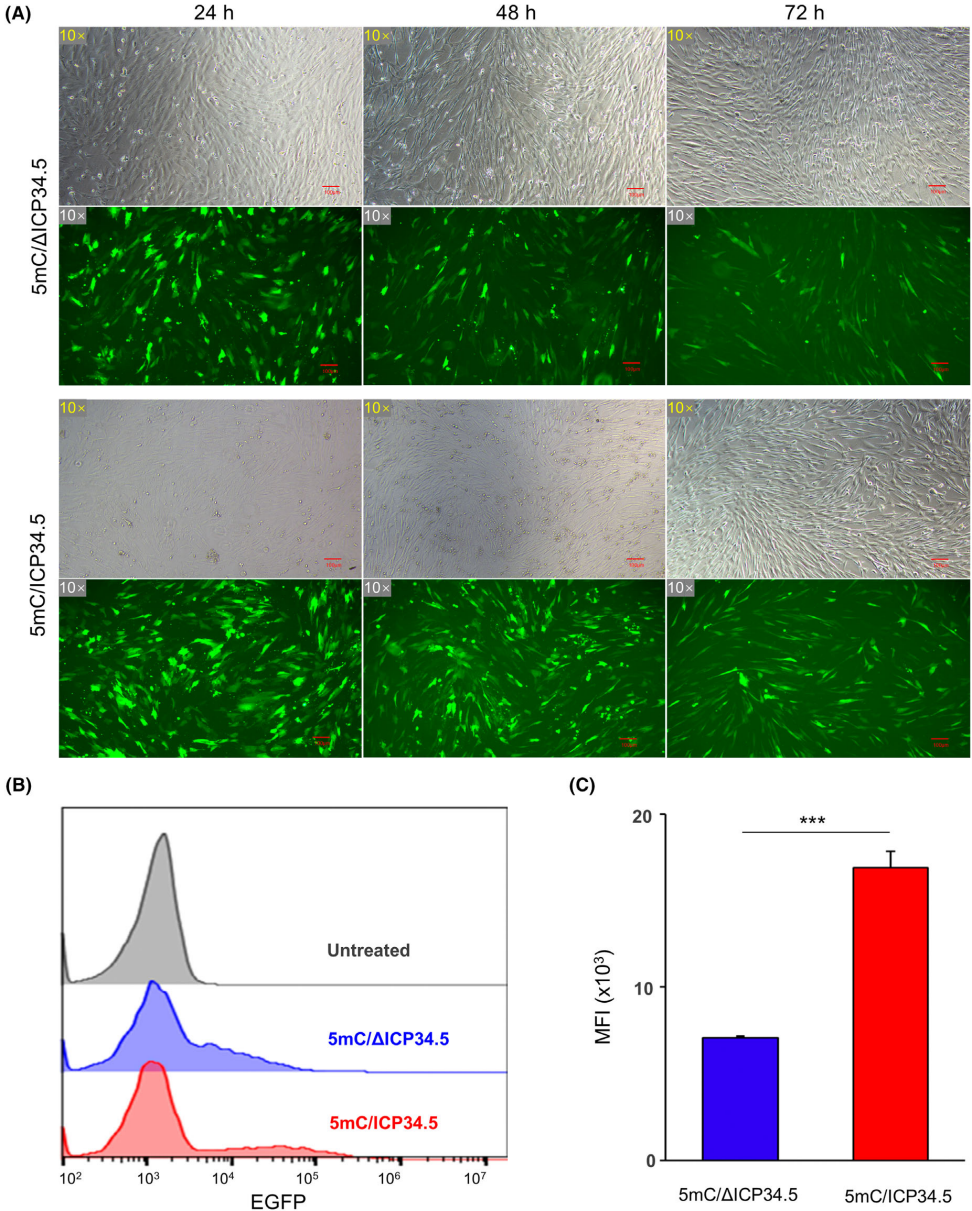
Intact ICP34.5 incorporating saRNA efficiently enhances EGFP expression in UC‐MSCs. (A) Representative fluorescence microscopy images of UC‐MSCs transfected with three types of saRNA at 24, 48, and 72 h. (B) Flow cytometry analysis of EGFP expression in UC‐MSCs transfected with saRNAs encoding either deletion‐mutated or intact ICP34.5 at 96 h. (C) Quantification of mean fluorescence intensity (MFI) of EGFP expression in UC‐MSCs transfected with saRNAs encoding either deletion‐mutated or intact ICP34.5, based on the flow cytometry analysis shown in (B). Statistical analysis was performed by Mann–Whitney *U* test. Data are means ± SD. *n* = 3. *P < 0.05, **P < 0.01, ***P < 0.001.

ICP34.5‐mediated dephosphorylation of eIF2α may enhance the translation efficiency of saRNA, thereby potentially increasing gene expression. To test this hypothesis, we transfected 293T cells with three types of saRNA. We observed that 5mC‐modified saRNA led to higher EGFP expression than the WT control, and co‐expression of ICP34.5 further increased EGFP levels (Fig. 3A,B). To confirm the role of ICP34.5 in gene expression enhancement in primary cells, we transfected UC‐MSCs with saRNA encoding either deletion‐mutated or intact ICP34.5. Consistently, saRNA‐encoding intact ICP34.5 resulted in higher EGFP expression and significantly elevated the mean fluorescence intensity (MFI) of EGFP compared with the deletion‐mutated group (Fig. 4A–C). Collectively, these results demonstrate that ICP34.5 significantly potentiates gene expression when delivered by the saRNA vector.

### saRNA‐encoding ICP34.5 attenuates innate immune responses

**Fig. 5:**
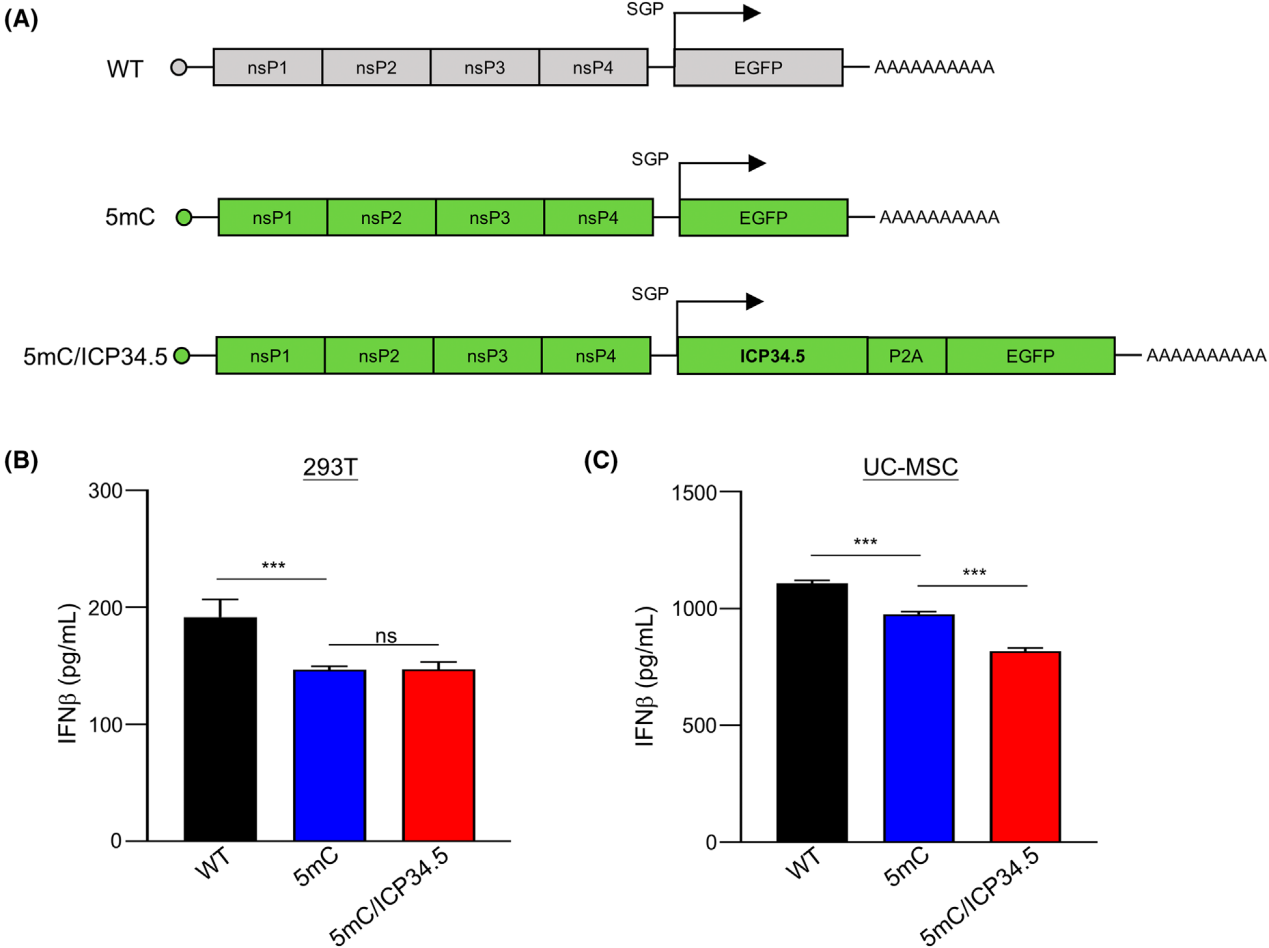
saRNA‐encoding ICP34.5 reduced innate immune responses in 293T and UC‐MSCs. (A) The structural schematic diagram of wild‐type saRNA and 5mC‐modified saRNA with or without encoding ICP34.5. (B, C) The secretion of IFNβ by saRNA‐transfected 293T cells or UC‐MSCs was assessed using ELISA. Statistical analysis was performed by Student's *t*‐test. Data are means ± SD. *n* = 3. *P < 0.05, **P < 0.01, ***P < 0.001, ns: not significant.

Previous study revealed that ICP34.5 also dampens IFN production by binding and sequestering TANK‐binding kinase 1 (TBK‐1), which phosphorylates IRF3 [32]. To assess the impact of ICP34.5 on Type I interferon expression induced by saRNA, we compared the protein levels of IFNα2 and IFNβ expression in 293T or UC‐MSCs transfected with saRNA encoding or not encoding ICP34.5 (Fig. 5A). Consistent with previous reports [20], the incorporation of 5mC in saRNA attenuated the secretion of IFNβ in both 293T and UC‐MSC (Fig. 5B,C). Compared with UC‐MSC cells, saRNA induces relatively lower levels of IFNβ in 293T cells, suggesting that the strength of the immune response induced by saRNA is cell‐type dependent (Fig. 5B,C). In accordance with our hypothesis, the saRNA‐encoding ICP34.5 further reduces the secretion of IFNβ by UC‐MSC cells (Fig. 5C). Nonetheless, we did not observe such a difference in 293T cells (Fig. 5B). We also assessed the secretion of IFNα2 and found that the expression levels of IFNα2 were comparably low in both 293T and UC‐MSC cell supernatants; the data are not shown. Collectively, these findings indicate that saRNA incorporating ICP34.5 significantly affects the innate immune responses. To investigate the impact of ICP34.5‐mediated suppression of the innate immune response on saRNA replication, we analyzed the mRNA levels of nsp4 and GFP in 293T and UC‐MSC cells following transfection with saRNA constructs incorporating or lacking ICP34.5. We found that the mRNA levels of nsp4 and GFP were comparable in both 293T and UC‐MSC cells (Fig. S1A and Fig. 1B). These results suggest that ICP34.5‐mediated attenuation of the innate immune response does not significantly affect saRNA replication. Therefore, the enhanced GFP MFI resulting from the expression of saRNA‐encoding ICP34.5 may be, at least in part, attributed to its suppression of interferon expression in the host cells.

## Discussion

The role of ICP34.5 in facilitating viral replication by countering host shutoff responses is well‐documented. Our findings extend this function to the enhancement of saRNA‐mediated gene expression, a potential advancement for vaccine applications. The increased transgene expression levels observed in the presence of intact ICP34.5 suggest a potential synergistic role in overcoming cellular barriers to foreign gene expression, thus improving the efficacy of gene delivery vectors.

ICP34.5 is a pivotal neurovirulence factor in HSV‐1, playing a critical role in viral replication and pathogenicity [32, 33]. This protein facilitates protein translation by bridging eukaryotic initiation factor 2a (eIF2a) and protein phosphatase 1 (PP1), a process essential for the PP1‐mediated dephosphorylation of eIF2α^28^. In our study, we conducted deletion mutations on the ICP34.5 binding site to eIF2α, confirming that saRNA effectively mediated eIF2α dephosphorylation and enhanced the expression of a fluorescent reporter gene only when encoding a fully functional ICP34.5. In addition to PP1, ICP34.5 protein can bind to a variety of host cell proteins to regulate the host cell's immune response. Consequently, we engineered saRNAs that either encode or do not encode ICP34.5 and compared their abilities to induce IFN production in host cells. We found that ICP34.5 can suppress IFN expression, with significant reductions in IFNα and IFNβ levels in 293T and UC‐MSC cells, suggesting that ICP34.5 inhibits IFN expression, and the lower levels of IFN might partially account for the reduced phosphorylation of eIF2α.

Dephosphorylation of eIF2α restores protein synthesis, implying that saRNA‐encoding ICP34.5 could enhance protein expression. We confirmed this in 293T cells with incomplete IFN signaling pathways. To further validate our hypothesis, we found that in UC‐MSC cells with intact IFN pathways, saRNA‐encoding ICP34.5 still upregulated the expression of the fluorescent reporter gene. These results confirm the advantage of ICP34.5 in enhancing the performance of saRNA vectors. Meanwhile, we also found that ICP34.5‐mediated attenuation of the host cell's innate immune response did not enhance saRNA replication. This suggests that the increased expression of GFP was not due to elevated GFP mRNA levels, but rather resulted from enhanced GFP translation driven by eIF2α dephosphorylation. A recent study identified saRNA with enhanced protein expression capabilities through macrodomain screening, which could reduce immunogenicity and enhance gene expression [34]. Therefore, the integration of ICP34.5 with other technological approaches may further refine the development of saRNA for vaccines and gene therapy drugs in the future.

ICP34.5 incorporating saRNA significantly suppresses the ability of UC‐MSC cells to secrete IFNβ, an effect not observed in 293T cells. Moreover, compared to 293T cells, saRNA induces significantly higher levels of IFNβ in UC‐MSC cells. This discrepancy may be attributed to differential response mechanisms of IFN across various cell types, necessitating further in‐depth investigation. It has been reported that plasmacytoid dendritic cells (pDCs) in peripheral blood mononuclear cells (PBMCs) are the primary cellular subset responsible for the production of type I IFNs in response to saRNA stimulation, indicating that the intensity of the innate immune response mediated by saRNA varies among different cell types [20]. However, we also observed that saRNA induced comparably low levels of IFNα2 expression in both 293T and UC‐MSC cells, suggesting that additional studies using other cell types are required to further validate the impact of ICP34.5 on IFNα2 expression. A study revealed HSV‐1 neurovirulence protein ICP34.5 interaction with mitochondrion‐associated factors, modulates mitochondrial dynamics and cellular stress responses [35]. Another study showed that ICP34.5 binds to Beclin 1 in mammals, inhibiting autophagy and potentially altering host cell survival and apoptosis [36]. Thus, further preclinical studies are necessary to optimize the enhancement of saRNA expression by ICP34.5 and to assess the long‐term safety and efficacy of this approach in animal models.

In conclusion, the results of this study could provide a significant advancement in the use of saRNA for gene therapy and vaccination, particularly in the context of neurotropic diseases where HSV‐1 ICP34.5 may have additional benefits.

## Conflict of interest

The authors declare no conflict of interest.

## Peer review

The peer review history for this article is available at https://www.webofscience.com/api/gateway/wos/peer‐review/10.1002/2211‐5463.70036.

## Author contributions

XL and YX: writing—original draft, visualization, and methodology. CZ, YW, and JZ: software, formal analysis, resources, and investigation. YX, YW, and SC: writing—review andediting, supervision, and funding acquisition.

## Declaration of generative AI and AI‐assisted technologies in the writing process

During the preparation of this work, the authors used Kimi in order to improve the readability and language of the manuscript. After using this tool, the authors reviewed and edited the content as needed and took full responsibility for the content of the published article.

## Supporting information


**Fig. S1.** ICP34.5 did not significantly enhance the replication of self‐amplifying RNA.

## Data Availability

The data that support the findings of this study are available from the corresponding author upon reasonable request.
