# Supplementary figures and images for: Co‐expression of HSV‐1 ICP34.5 enhances the expression of gene delivered by self‐amplifying RNA and mitigates its immunogenicity
Source: FEBS Open Bio. 2025 Apr 9;15(7):1079–89. doi: 10.1002/2211-5463.70036 (PMC12226409; doi:10.1002/2211-5463.70036)

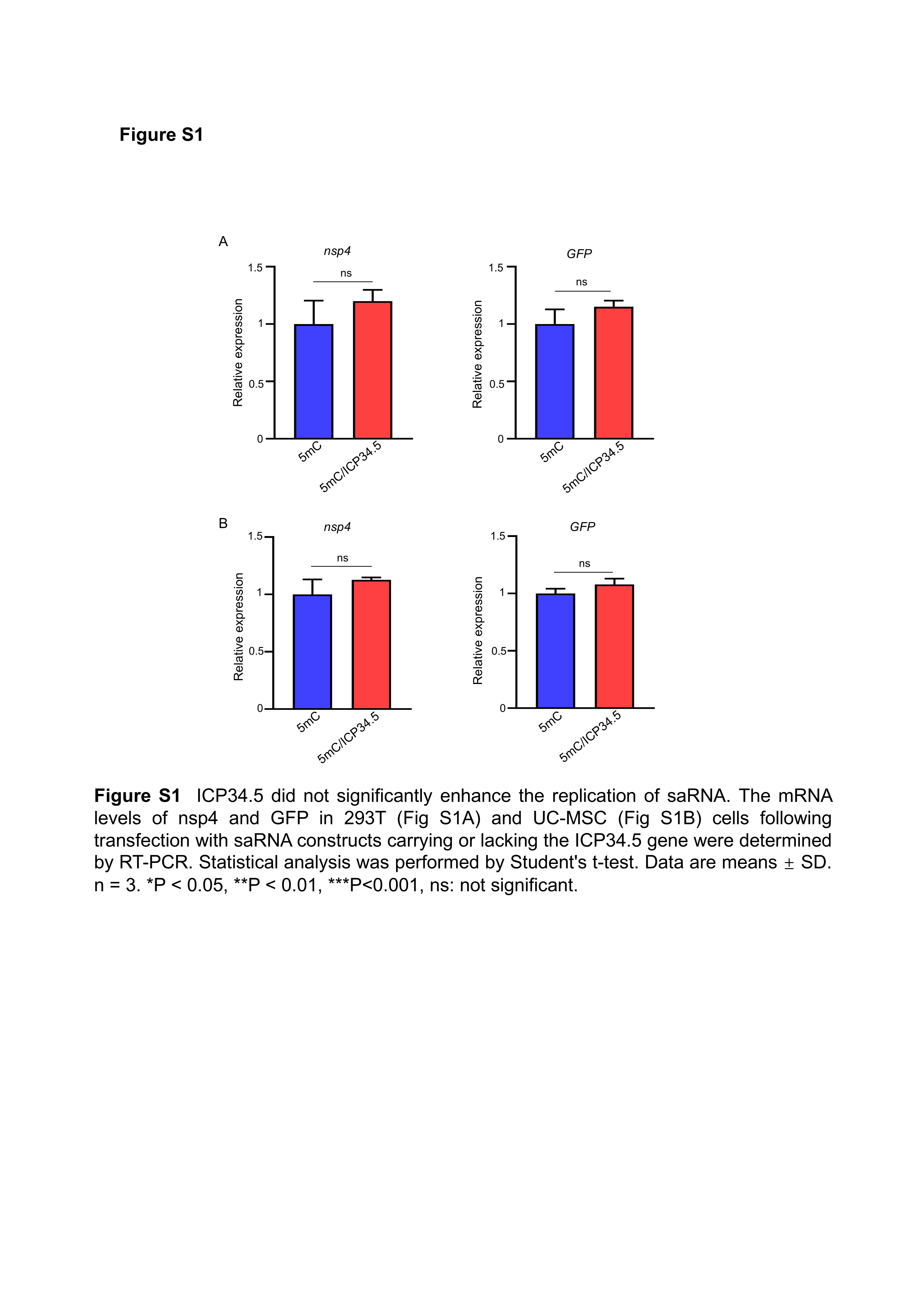

Supplement: Supplementary file 1 — Fig. S1. ICP34.5 did not significantly enhance the replication of self‐amplifying RNA. [file FEB4-15-1079-s001.tif]
